# Systolic and diastolic ventricular function in zebrafish embryos: Influence of norepenephrine, MS-222 and temperature

**DOI:** 10.1186/1472-6750-8-21

**Published:** 2008-02-27

**Authors:** Martin A Denvir, Carl S Tucker, John J Mullins

**Affiliations:** 1 Centre for Cardiovascular Science, Queen's Medical Research Institute, University of Edinburgh, Edinburgh, Scotland, EH16 4TJ, UK

## Abstract

**Background:**

Zebrafish are increasingly used to study the influences of gene mutation and manipulation on cardiac development, structure and function. In this study, a video edge detection system was used to characterise, continuously, cardiac ventricle function in 2–5 days old zebrafish embryos embedded in 0.6% agar and examined under light microscopy at room temperature (22°C). Using video edge detection software (IonOptix Inc), the motion of a small region of the cardiac ventricle wall was converted to a continuous chart trace allowing analysis of wall motion amplitude (WMA) and myocardial wall velocity during systole (MWVs) and diastole (MWVd).

**Results:**

Cardiac wall motion characteristics changed progressively from day 2 to 5 (WMA, 2-days, 17.6 ± 4.4 μm vs 5-days, 24.6 ± 4.7 μm, p < 0.01). MWVd was more rapid than MWVs at all developmental time points. Embryonic hearts were also assessed after increasing concentrations of norepenephrine (NE) and the anaesthetic agent MS222 (tricaine) were added to the bathing water. In response to NE, WMA increased significantly more in 4 day embryos compared with 2 day embryos (change in WMA,13.6 ± 8.2 μm vs 4.0 ± 8.8 μm, p = 0.01, respectively) while the decrease in WMA in response to MS222 was similar in both 2 and 4-day embryos. Heart rate, MWVs and MWVd were significantly higher at 28°C compared with 22°C. No differences in cardiac function were observed between AB and Golden strains.

**Conclusion:**

Video edge detection appears sufficiently sensitive to detect subtle changes in diastolic and systolic cardiac function during development and changes resulting from pharmacological and environmental interventions. Such measurements could be valuable in assessment of altered cardiac function after genetic manipulation.

## Background

In the short period that the zebrafish (*Danio rerio*) has been utilised for biomedical studies it has emerged as a powerful genetic tool and an ideal model system for both organogenesis and as a vertebrate model for human disease [1]. The embryonic zebrafish heart is the first organ to develop, and shows similarity to the embryonic human heart, specific comparison has been drawn to show developmental parity between the 24 hours post fertilisation (hpf) zebrafish heart and the human heart at three weeks [2,3]. One clear advantage of utilising the zebrafish, as a model organism, is that normal cardiac function in embryonic zebrafish is not required for several days, sufficient oxygen is obtained by diffusion [4]. Fish, unlike rodent models, are not dependent on a cardiovascular system for survival during embryogenesis. Cardiac activity is not coupled with metabolic demand [5] and hence defects in cardiac development or function are less likely to result in early mortality. Furthermore, the zebrafish embryo is virtually transparent which permits imaging of internal organs, especially the heart, using standard light microscopy. For these reasons, the zebrafish is an ideal model system in which to examine developmental biology and organogenesis.

The zebrafish heart is essentially two chambered with valves dividing the atrium and ventricle and a second valve separating the ventricle from the outflow tract or bulbous arteriosum [6]. Prior to 22 hpf the lateral cardiac primordia join to form a definitive heart tube which begins to contract in a coordinated, rhythmic fashion and by 24 hpf contributes to circulation by peristaltic waves. At 36 hpf morphological differentiation from the heart tube to chambers is observed and by 72 hpf the circulatory system is more mature with the heart having looped to form a dorsally positioned atrium [7]. All major organs are present by this stage. By 5-days post fertilisation (dpf) the embryonic heart is essentially that of an adult teleost configuration [7,8] with a functional cardiovascular system conforming to a vertebrate developmental plan [9].

The zebrafish embryo measures approximately 3.5 mm in length at 3 dpf. This small size presents a technical challenge to assessment of the functional characteristics of the heart. Unsurprisingly therefore, many previous studies have focused predominantly on counts of heart rate observed directly by microscopy or combined with video image capture [10-12]. These studies have limitations in that simple measures of heart rate do not provide information on cardiac contractility. Other studies have reported estimates of cardiac output and ejection fraction using single systolic and diastolic video frames from light microscopy to derive cardiac output and ejection fraction [13]. Such estimates are limited in that they are derived from 2-dimensional images using geometric assumptions of ventricular shape and they reflect systolic function only; there are no reports of measures of diastolic function in the zebrafish. More recently reported techniques using rotational imaging [14] and confocal microscopy [15] provide detailed three dimensional imaging of the zebrafish embryonic heart but this is not widely available and is relatively expensive and time consuming to perform. Furthermore, other than counts of heart rate, the effects of pharmacological interventions on cardiac systolic and diastolic function have not been widely studied.

The aim of this study was to assess and implement a technique which would allow the continuous assessment of heart rate, ventricular wall motion amplitude and wall motion velocity in systole and diastole. This technique potentially allows the accurate assessment of both the extent of response and the time course of responses to various physiological and pharmacological interventions and could be adapted to high throughput screening.

## Results

### Cardiac wall motion changes during development (days 2 to 5)

Table 1 summarises the baseline characteristics of heart rate, myocardial wall velocity in systole (MWVs), myocardial wall velocity in diastole (MWVd) and wall motion amplitude (WMA) for zebrafish embryos ranging from 2 to 5 dpf. The 2-day embryos had significantly lower heart rates, amplitude of wall motion and significantly lower MWVs. MWVd was also lower but this did not reach statistical significance. Of note, and not previously reported, the rate of ventricular relaxation, as assessed by MWVd, is significantly faster than ventricular contraction (MWVs) at all stages of development examined in this study.

**Table 1:** comparison of baseline heart rat, wall motion amplitude (WMA), myocardial wall velocity in systole (MWVs) and myocardial wall velocity in diastole (MWVd) at 22°C in Golden zebrafish embryos 2 to 5-days post-fertilisation (Values are mean ± (SD))

	2 days (n = 6)	3 days (n = 8)	4 days (n = 7)	5 days (n = 6)	ANOVA
Heart rate (bpm)	141 ± 14.2	147.2 ± 20.9	165.90 ± 16.8	171.5 ± 12.9	<0.001
MWVs (μm/s)	195.4 ± 54.3	207.6 ± 61.6	264.6 ± 75.7	275.6 ± 154.6	0.03
WMA (μm)	17.6 ± 4.4	18.2 ± 4.5	26.7 ± 7.4	24.6 ± 4.7	0.01
MWVd (μm/s)	262.2 ± 101.9	268.9 ± 108.2	402.8 ± 133.7	345.8 ± 95.0	0.09

### Effects of norepenephrine

**Figure 1:**
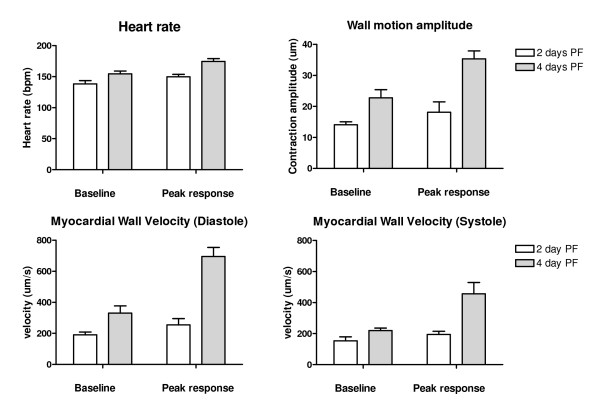
Peak responses to norepenephrine in 2-day (n = 6) and 4-day (n = 6) zebrafish embryonic hearts (values are mean ± SD).

Norepenephrine significantly increased heart rate, myocardial wall velocity in systole, myocardial wall velocity in diastole and wall motion amplitude in 2 and 4 day embryos (Figure 1). The observed changes in these parameters were less marked in 2 day compared with 4 day embryos (Table 2). In 2 dpf embryos, NE produced a mean 20% increase in MWVs and a mean 15% increase in MWVd. However, at 4 dpf NE produced a mean 75% increase in MWVs and a mean 90% increase in MWVd.

**Table 2:** peak change from baseline in heart rate, wall motion amplitude (WMA), myocardial wall velocity in systole (MWVs) and myocardial wall velocity in diastole (MWVd) in response to norepenephrine: 2-day vs 4-day embryos (Golden strain)

	**2-days (n = 5)**	**4-day (n = 7)**	P*
Heart rate (bpm)	+11.6 ± 7.2	+19.9 ± 7.3	0.07
WMA (μm)	+4.0 ± 8.8	+13.6 ± 8.2	0.07
MWVs (μm/s)	+41.7 ± 88.8	+184.9 ± 126.1	0.05
MWVd (μm/s)	+42.6 ± 75.6	+364.7 ± 139.9	<0.001

*comparison between 2 and 4 day embryos

### Effects of MS222 (Tricaine)

**Figure 2:**
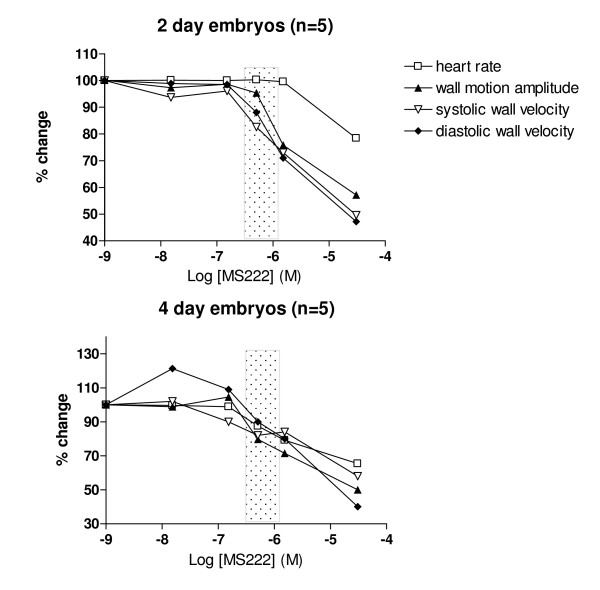
Effects of increasing concentrations (M, moles)   of anaesthetic agent MS222 on cardiac function in 2 and 4-day embryo hearts (shaded area represents typical range of concentrations of MS222 used for fish anaesthesia).

Administration of increasing concentrations of MS222 to the bathing water resulted in progressive reduction in heart rate, wall motion amplitude and myocardial wall velocities in systole and diastole in both 2 day and 4 day embryos (Figure 2). In 2 day embryos, wall motion amplitude, myocardial wall velocities in systole and diastole fell by approximately 30% before any significant reduction in heart rate was observed. This occurred over a concentration range of MS222 normally used in our laboratory for anaesthesia (70–100 mg/litre). In 4 day embryos, there was a slight rise in the mean myocardial wall velocity in diastole at low doses of MS-222 accompanied by a small rise in heart rate (Figure 2). At higher doses the heart rate fell in conjunction with other wall motion parameters.

### Effects of temperature on zebrafish cardiac function

Heart rate was significantly higher at 28°C compared with 22°C (Table 3). Wall motion amplitude did not change significantly at higher temperature but there was a significant increase in myocardial wall velocity in systole and a borderline significant increase in myocardial wall velocity in diastole.

**Table 3:** effects of temperature on baseline heart rate, wall motion amplitude (WMA), myocardial wall velocity in systole (MWVs) and myocardial wall velocity in diastole (MWVd) in 3 day Golden embryos (n = 6) at 22°C and 28°C (values are mean ± SD).

	**Temperature**		
	
	**22°C**	**28°C**	**P**
**Heart rate (bpm)**	136.2 ± 8.3	276.2 ± 22.0	<0.001
**MWVd (μm/s)**	265.2 ± 66.1	364.9 ± 117.2	0.07
**MWVs (μm/s)**	172.6 ± 40.3	316.3 ± 89.5	0.02
**WMA (μm)**	17.2 ± 3.6	15.6 ± 3.5	0.46

### Effects of zebrafish strain (Golden vs. AB)

Comparing AB and Golden strains at 3 days and 5 days, there were no significant differences observed in the any of the wall motion characteristics at these developmental time points (data not shown).

## Discussion

This study has applied a video-edge detection technique previously validated in assessing motion in mammalian single cardiac myocytes [16]. The technique provides continuous measurement of a number of cardiac parameters which reflect different aspects of cardiac function: heart rate, amplitude of myocardial wall movement, myocardial wall velocity during systole and myocardial wall velocity during diastole. Assessing these parameters during the first 5 days of development has revealed a progressive increase in heart rate and progressive improvement of wall motion characteristics at 24 hour intervals. Furthermore, we have demonstrated clear changes in myocardial wall velocities in systole and diastole in response to drugs administered into the bathing water and changes resulting from alteration in bathing water temperature. The findings therefore not only suggest that this technique is feasible but that it has the capacity and sensitivity to detect both large and small differences in cardiac function which could result from genetic or environmental manipulation.

The technique is also useful in determining responses to drugs that are known to influence cardiac function in fish. Drugs such as norepenephrine appear to be readily absorbed through the gills even with the embryo embedded in a weak agar solution. Other workers have reported similar responses to cardio-active drugs when introduced into the bathing water [17-19]. This pharmacological approach provides the potential for experiments examining the responsiveness of zebrafish myocardium to cardio-active drugs following genetic manipulations which potentially induce phenotypes analogous to human cardiomyopathy or heart failure [20,21]. In this way, altered responsiveness to such drugs could be useful in detecting and further defining similarities in phenotype to human disease.

The findings of this study also provide insights into developmental time points of the adrenergic system in the zebrafish heart. While norepenephrine had a significant effect on 4-day zebrafish embryo hearts this was much less obvious in 2-day embryos. The difference in response to norepenephrine was most marked for relaxation velocity which is known to be strongly influenced by adrenoreceptor mechanisms in mammalian tissue. This is consistent with previously published work in zebrafish showing response of heart rate to isoproterenol [19] at various developmental time points and work showing an increase in cardiac out put in 3 and 4-day zebrafish embryos injected with norepenephrine [17]. The ability to assess cardiac chamber wall motion directly provides more specific information on the response of the heart to drugs compared with techniques focusing on heart rate alone. Such studies have reported bradycardia in response to drugs known to prolong repolarisation [18] but there are also reports of bradycardia in response to drugs not known to act directly on cardiac conducting mechanisms, such as warfarin and atorvastatin [12]. This highlights the need for characterisation of drug responses in the zebrafish heart using techniques that assess not only heart rate but also chamber specific wall motion.

In contrast to norepenephrine, the anaesthetic agent MS222 was found to depress cardiac function in a dose dependent fashion in 2 and 4-day embryos. This suggests that the mechanism of action of this drug on the heart is established as early as 48 hours after fertilisation. Previous studies have described a decrease in heart rate at higher concentrations of MS222 and a slight increase at lower concentrations [22]. One previous study suggested a reduction in stroke volume and cardiac output measured indirectly using aortic blood flow [23]. Previous studies have described greater sensitivity to MS222, in terms of lethality and deep anaesthesia, at younger gestation [24]. Our study goes further and suggests that significant reductions in cardiac wall motion indices occur in 2-day embryos even before any change in heart rate is observed. This important finding raises the possibility that there is significant depression of cardiac contractility at concentrations of MS222 widely used for fish anaesthesia in younger embryos. In the study reported here, all assessment of cardiac wall motion comparing 2 – 5 day embryos was conducted without anaesthesia with the fish immobilised in agar.

The effect of temperature on zebrafish heart rate has been examined previously in normal zebrafish [25] and in the zebrafish *breakdance *mutant [11]. Heart rate appears to show a steep positive association with temperature with rates of up to 260 beats per minute reported at 31°C. In this study we have shown a similar steep relationship between temperature and heart rate and we have also demonstrated, for the first time, significant increases in the velocity of the myocardial wall during contraction and a trend for enhanced velocity during relaxation at higher temperatures. This confirms a close coupling between myocardial metabolism and temperature and supports the need for careful temperature control during experiments assessing cardiac function in poikilothermic fish.

There were no differences observed between AB and Golden strains of zebrafish comparing 3 day and 5-day embryos. AB strain heart rates found in this study are similar to those found by other workers, however, a wide range for heart rates has been reported ranging from 140–190 beats per minute at 3-days to 135–230 beats per minute at 5-days [22,26]. Different heart rates have been reported in Albino and AB strains [17,19] but this is the first report comparing different strains using qualitative assessment of heart rate and cardiac wall motion.

Screening large numbers of zebrafish embryos is a strategy widely used to identify phenotypic features suggestive of an underlying genetic defect [1,10,27]. This video edge detection technique could be applied to examine more sensitively for cardiac wall motion abnormalities in such screens. However, the technique does require a degree of operator skill and it is likely that the number of fish that could be assessed per day would be approximately 30–40. It is possible that the technique could be combined with more automated microscopic systems, using wells with individual fish to provide more rapid assessment of ventricular wall movements. While this would be valuable, the technique also lends itself to the evaluation of qualitative differences in cardiac function at specified developmental time points and could be used in knock down experiments [28] of genes known to affect cardiac performance.

## Conclusion

This study has outlined a novel method for assessing cardiac wall motion in zebrafish embryos from 2-days to 5-days post-fertilisation. The technique extends our ability to assess cardiac function from, simple measures of heart rate and snap-shot assessments of changes in ventricular area using single frame images, to continuous assessment of wall motion. The technique appears sensitive to pharmacological and physiological interventions and provides a relatively inexpensive method of characterising subtle and cycle-specific changes in cardiac function resulting from genetic and pharmacological manipulation.

## Methods

### Husbandry and maintenance

A zebrafish (*Danio rerio*) Golden (B1) strain was used throughout this study except in one experiment where cardiac motion characteristics in this Golden strain were compared with the AB strain. Fish were maintained at 28.5°C under standard conditions [29,30] and unless stated the experimental protocols were conducted at room temperature (22°C).

Embryos were collected from random Golden matings and when correctly aged were anaesthetised (20 μM buffered MS-222, Sigma) and if required embryos were mechanically removed from their chorion. Embryos were then embedded in 2 ml 0.6% SeaKem LE agarose (Cambrex) and orientated carefully in a lateral position to optimise visualisation of the heart. Following agar-embedding, fish were allowed to recover in fresh system-water (3 ml) with no anaesthetic for a minimum of 30 mins, this in turn was replenished before the commencement of any experiment. Agar embedding is not considered to have detrimental effects on zebrafish embryo physiology [22].

### Video edge-detection system

SoftEdge™ (IonOptix Corporation) measures real-time length via contrast analysis of digitized image data. A frame-grabber card mounted in the acquisition computer continuously digitizes the output of the video camera and displays it on a monitor. The video camera was mounted on a light microscope (Axioskop II MOT compound microscope) with a ×40 water dipper objective, setup for maximum differential interference contrast.

**Figure 3:**
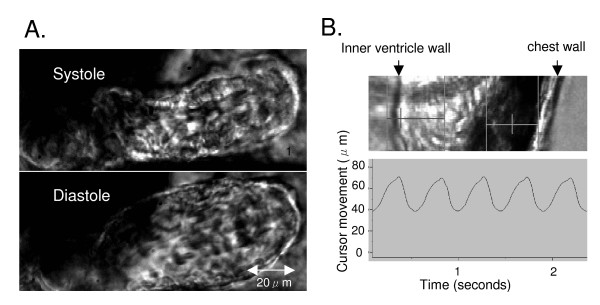
2-day zebrafish embryo ventricle in systole and diastole (panel A). Inner ventricle wall is tracked by a cursor (panel B, left vertical line) and the movement displayed a continuous trace (panel B, contraction is upwards).

The user selects video lines on the edge of the heart corresponding to the ventricular wall using a cursor (Figure 3). Edge detection is based on either image intensity or the derivative of image intensity. Edges are detected from "outside in" and image contrast is enhanced using the video gain and offset controls of the frame grabber PC card. Optimal edge contrast is achieved by adjusting the settings on the microscope and the camera. The user specifies a threshold value and a crossing condition. The computer analyses each video image (see typical image in Figure 3, panel A) and treats the crossing of the threshold value as the edge location. This signal is displayed as a continuous trace (Figure 3, panel B). The sampling frequency of the camera was set at 120 Hz to allow optimal temporal resolution. The signal was calibrated using a standard millimetre graticule and values are expressed as micrometers (wall motion amplitude) or micrometers per second for myocardial wall velocity in systole (MWVs) and diastole (MWVd).

### Baseline assessment of cardiac wall motion during early development

Baseline measurements of ventricular wall motion were observed in zebrafish embryos ranging in age from 2 to 5 dpf at 22°C. The wall tracking cursor was placed in a position to provide an optimal trace of wall motion and allowed to stabilise over a period of 10–20 minutes. When a stable trace with good myocardial wall tracking could not be obtained then this fish was discarded and the next assessed. Successful and consistent assessment of myocardial wall tracking was obtained in 80% or more embryos. In 2 and 3 day embryos wall motion was assessed in the outer wall at the mid-point of the ventricle close to the pericardium where a consistent and stable trace was readily obtained. In 4 and 5 day embryos, after the heart had undergone full looping, the most stable and consistent site to obtain a trace of the cardiac wall motion was on the outer wall close to the atrium. Repeated separate measurements of wall motion and velocity over a period of 15 minutes indicated no more than a 5% variation in each of the measured parameters.

To assess the effects of agar-embedding on cardiac function, heart rate and wall motion were assessed in agar-embedded and non-embedded 2 day embryos at baseline and after the addition of 100 mg/ml of MS222. Embedding in agar had no significant effect on the rate of onset of MS222-induced cardiac depression with maximum effect seen 10–15 minutes after MS222 was added to the bathing water (data not shown).

### Pharmacological interventions

Heart rate, amplitude of ventricular wall movement, wall motion velocity in diastole and systole were observed at baseline and after increasing concentrations of norepenephrine and MS222 (Sigma chemicals) were added to the bathing water. These drugs were chosen to assess whether the technique was sensitive to both increases and decreases in cardiac performance resulting from pharmacological intervention.

In 2 day and 4 day embryos, norepenephrine was added to a standard volume of bathing water using a micropipette to give known final concentrations ranging from 10^-9 ^M to 10^-3 ^M. Cardiac wall motion and heart rate were assessed continuously throughout this period using the video edge detection system. 10 representative cardiac cycles were analysed 10 minutes after each concentration of the drug was administered. This time period was chosen after pilot experiments were performed assessing the time-lag between adding the drug to the bathing water and the onset of a measurable effect on cardiac contraction and relaxation. At the end of this period the bathing water was refreshed 2–3 times and a new baseline established. A similar protocol was repeated using MS222 (10^-6 ^M to 10^-3 ^M) while continuously monitoring heart rate and ventricular wall motion.

### Effects of temperature on wall motion and heart rate

3-day embryos (Golden B1 strain) were embedded in agar in a dish placed on an electronically controlled heated microscope stage (Bioptech, Delta T open dish system). Heart rate and cardiac wall motion were assessed at a baseline temperature of 22°C. The stage was then progressively heated to 28°C and then maintained at this temperature for a further 1 hour. Heart rate and cardiac wall motion were reassessed.

### Comparison of baseline heart rate and cardiac wall motion in Golden and AB strains

3 day and 5 day zebrafish embryos derived from Golden and AB strains were embedded in agar and their heart rates and cardiac wall motion assessed at 22°C. No pharmacological or temperature interventions were performed on these fish.

### Statistical analysis

Values represent an average of 10 cardiac cycles for each set of conditions in individual zebrafish embryo hearts. Data are presented as means ± standard deviation (SD). A 2-way repeated measures ANOVA was used to compare means within and between groups. Where applicable, Tukey's multiple comparison procedure was performed to assess for specific pair-wise comparisons post hoc. Significance was accepted at the 5% level (p = 0.05).

## Authors' contributions

MD conceived of the idea for the study, co-designed the study, performed the experiments and co-wrote the manuscript. CT also contributed to the study design, prepared the zebrafish embryos, helped performed the experiments and co-wrote the manuscript. JJM helped design the experiments, provided advice and criticism during the experiments and helped write the manuscript. All authors read and approved the final manuscript.
